# Does the use of protective face masks affect temporomandibular joint function?

**DOI:** 10.12669/pjms.41.2.10588

**Published:** 2025-02

**Authors:** Sena Ozdemir Gorgu, Yasin Yildirim, Pınar Kaya, Gizem Ergezen, Eda Uzuner

**Affiliations:** 1 Sena Ozdemir Gorgu, PT, Ph.D. Department of Orthotics and Prosthesis, Faculty of Health Sciences, Istanbul Medipol Universty, Istanbul, Turkey; 2 Yasin Yildirim Department of Physical Therapy and Rehabilitation, Faculty of Health Sciences, Istanbul Gedik University, Istanbul, Turkey; 3 Pınar Kaya Department of Physical Therapy and Rehabilitation, Faculty of Health Sciences, Istanbul Medipol Universty, Istanbul, Turkey; 4 Gizem Ergezen Department of Physical Therapy and Rehabilitation, Faculty of Health Sciences, Istanbul Medipol Universty, Istanbul, Turkey; 5 Eda Uzuner Department of Speech and Language Therapy, Faculty of Health Sciences, Istanbul Medipol Universty, Istanbul, Turkey

**Keywords:** Masseter muscle, N95 face masks, Surface electromyography, Temporomandibular joint disorders

## Abstract

**Objective:**

Long-term mask use can trigger or exacerbate various health issues. This prospective experimental study evaluated the impact of protective face masks on masseter muscle activity and temporomandibular joint (TMJ) function reported by participants.

**Methods:**

This prospective, experimental tudy was conducted to investigate the effect of face masks on TMJ function, we used surface electromyography to assess masseter muscle activity at rest, during maximum contraction of the masseter muscle, and while reading a text. We also evaluated the intensity of the masseter muscle pain using an algometer. The participants underwent these evaluations at the Istanbul Medipol University between July and October 2022.

**Results:**

The study included 24 female participants with an average age of 28.5±5.40 years. After at least five hours of daily N95 mask use, a significant increase in masseter muscle activity was observed at rest and during speech (p=0.01; p=0.04, respectively). However, no significant changes in the maximum contraction of masseter muscle or pain threshold were observed (p>0.05).

**Conclusion:**

This study found that the use of N95 mask is associated with restricted TMJ movements and discomfort, and an increase in its use is associated with restricted TMJ movements, discomfort, and increased masseter muscle activity. Questions regarding mask-wearing-related habits should be included in the routine lifestyle assessment of patients who report TMJ complaints.

## INTRODUCTION

Infectious diseases have affected society throughout history. These diseases are caused by bacteria or viruses, and are transmitted through contact or droplets.^1^ Masks protect individuals from disease.^2^ N95 and surgical masks are commonly used to prevent respiratory diseases. For effective protection, it is important to ensure that masks properly cover the nose, mouth, and chin.^3^

Temporomandibular joint disorders (TMDs) can cause pain in the chewing muscles and peripheral muscles, limit mandibular movements, cause functional loss, and produce joint sounds.^4^ A recent systematic review has revealed that both task and muscle specific changes in masticatory muscle activity are evident in adults with TMDs compared with healthy controls.^5^ In particular, it has been demonstrated that these changes have a greater impact on the masseter and temporalis muscles.^6^ The significance of psychosocial factors in the development and maintenance of TMDs is widely acknowledged, and there is a high prevalence of psychological disorders among patients with TMDs.^7^ TMDs are common in the adult population, with a higher prevalence observed in young women aged 18–45 years.^8^ Based on clinical observation, there has been an increase in the number of cases of TMDs in the recent decades.^9^

The coronavirus disease pandemic may have increased bruxism and TMDs due to stress; however, temporomandibular joint (TMJ)-related pain and dysfunction from pandemic stress remain unstudied.^10^ The study aimed to evaluate the effect of the use of protective face masks on TMJ function and masseter muscle activity.

## METHODS

This was a prospective, experimental study. The study was conducted in accordance with the Declaration of Helsinki and approved by the registration number NCT06103851 (www. clinicaltrials. gov). All participants provided written consent through a “Subject Informed Consent Form.”

### Ethical Approval:

It was obtained from the Institutional Review Board of the Istanbul Medipol University (approval number: E-10840098-772.02-4611) on September 21, 2021.

Individuals who agreed to participate in the study after being informed of its objectives and characteristics were included upon approval of the informed consent form. The participants were assessed at the Istanbul Medipol University Center for Speech and Swallowing Therapy and Innovative Technologies Research and Application between July and October 2021. Female individuals aged 18–45 years without orthopedic, rheumatic, or neurological problems related to the jaw were included in the study, whereas those with skin and/or lung diseases, an inability to use protective face masks for an extended period, and pregnant individuals were excluded. The power of the test was calculated using G*Power 3.1. The power analysis was applied post-hoc for Wilcoxon Signed-Rank Test using the maximum contraction (max cont) value differences (Power (1-β err prob) = 0.912).

### Outcome Measures:

At the beginning of the study, the research questions were determined through a literature review conducted by the researchers and by obtaining opinions from expert clinicians. To investigate the impact of protective mask use on the TMJ during the pandemic, a descriptive personal information form consisting of 11 questions was created. Each participant was informed about the study and asked to respond anonymously to a free online survey. The first section of the survey provided a brief overview of the study’s title and purpose. Information regarding the participants’ age, height, weight, and sex was collected. In the second section, the participants were asked about TMJ complaints related to the use of protective masks.

### Surface Electromyography (sEMG) Measurements:

In the literature, sEMG is commonly used to diagnose TMDs and assess muscle cell activation, tension, and fatigue.^11^ Participants’ sEMG measurements were taken in a stable resting position, during maximum contraction of masseter muscle, and while reading a text that included oral sounds, utilizing the VitalStim® Plus Electrotherapy System sEMG modality.

### Assessment Protocol:

Participants were provided with masks of the same brand and size, free of charge. This was due to the preference for N95 respirators over surgical masks in protecting against viral respiratory pathogens.^12^ The first measurement was conducted in the morning without mask usage (T0 = Before using any masks during the day; unmasked), and the second measurement, including at least five hours of mask usage after the initial assessment, was performed with a mask on (T1 = Measurement after at least five hours of mask use; with mask).

During the T0 measurement, the participants were instructed to sit in an upright and comfortable position on a chair in a restful posture without wearing a protective mask in the morning. The resting muscle activity was recorded once for 30 s (T0 rest). For another measurement, the participants were instructed to clench their jaw as strongly as possible in the intercuspal position and maintain the maximum level of contraction for five seconds (max cont.). The average work sEMG data of the masseter muscle were recorded (T0 max cont).

In the measurement to determine the activity of the masseter muscle during speech, the “Morning Surprise” text, found in the Nasometric Assessment Device, which was created by adapting the Simplified Nasometric Assessment Procedures Test-R developed by Ann Kummer for assessing resonance disorders into Turkish was used.^13^ During the participants’ reading of the text containing oral sounds, the average sEMG signal from the masseter muscle were recorded (T0 speech). For the T1 timepoint (minimum: five hours; maximum: six hours), the participants were asked to use the N95 mask for at least five hours throughout the day, and the measurements were repeated with a mask, at rest, during maximum contraction, and during speech (T1 rest, T1 max cont, T1 speech). All sEMG measurements were repeated three times at 30-s intervals for both the right and left masseter muscles, and the average values were recorded.

### Pressure Pain Threshold Assessment:

Measurements of the masseter muscle were taken by a physiotherapist using a hand algometer (Baseline® algometer, Fabrication Enterprises Inc., White Plains, NY, USA) and recorded. Examination form at timepoints T0 and T1. Measurements were performed three times at 60-s intervals and the average value was accepted as the pain threshold.

### Statistical Analysis:

The data obtained were analyzed using the Statistical Package for Social Sciences for Windows 22.0. Descriptive statistical methods such as means, and standard deviations were used for data evaluation. The difference between pre and post mask use measurements was analyzed using the Wilcoxon Signed-Rank Test.

## RESULTS

The study included 24 female participants who met the inclusion criteria, with an average age of 28.5 ± 5.40 years and a body mass index of 21.25 ± 2.69 kg/m^2^. The first two questions were scored out of 10 points. Overall, 80% of the participants reported that they experienced pain in the jaw joint in their daily lives before the pandemic rated at or below three points out of 10 points (52.5%; zero points, 8.3%; one point, 8.3%; two points, 4.2%; two points); this rate was at a similar level after long-term mask use (54.2%; zero points, 4.2%; one point, 16.7%; two points, 8.3%; two points).

In addition, in the last question of the survey regarding TMJ complaints after the long-term use of a protective mask, they stated that they felt cramped (n = 9, 37.5%), experienced limitation of movement (n = 5, 20.8%), clenching of the joint (n = 6, 25%), and experienced pain (n=3, 12.5%), whereas 37.5% reported having no complaints. The results of the eight questions survey form provided to the participants, which they were asked to respond to on a five points Likert scale, aiming to determine TMJ complaints and complaints regarding the use of a protective face mask, are shown in Table-I. A comparison of the masseter muscle activity values at rest, during maximum contraction, and during speech obtained at T0 and T1, as well as the pressure pain threshold measurements of the masseter muscle is shown in Table-II.

**Table-I:** Questions and answers for a survey form.

	I absolutely agree	I agree	I partially agree	I do not agree	I absolutely disagree
1. Did you experience any jaw joint discomfort in your daily life before the pandemic, before using a mask for protection?	2 (8.3%)	1 (4.2%)	4 (16.7%)	8 (33.3%)	9 (37.5%)
2. Did you experience any jaw joint discomfort after wearing a mask for a long time during the pandemic?	1 (4.2%)	0	7 (29.2%)	8 (33.3%)	8 (33.3%)
3. Do you experience stiffness in your jaw or difficulty moving after wearing a mask?	1 (4.2%)	0	8 (33.3%)	8 (33.3%)	7 (29.2%)
4. Does long-term use of masks increase your stress level?	6 (25%)	10 (41.7%)	6 (25%)	1 (4.2%)	1 (4.2%)
5. Does wearing a mask restrict your mouth movements?	5 (20.8%)	12 (50%)	2 (8.3%)	3 (12.5%)	2 (8.3%)
6. Does the mask limit your ability to move your mouth during speech?	8 (33.3%)	10 (41.7%)	5 (20.8%)	1 (4.2%)	0
7. Does wearing a mask give you a headache?	6 (25%)	11 (45.8%)	5 (20.8%)	1 (4.2%)	1 (4.2%)
8. Do the threads of the mask feel like they are tugging on your ears?	3 (12.5%)	10 (41.7%)	6 (25%)	3 (12.5%)	2 (8.3%)

**Table-II:** Comparison of masseter sEMG measurement values and pressure pain threshold results during rest, maximum contraction and speech.

Masseter	N	T0 (Avg±SD)	T1 (Avg±SD)	p value (T0-T1)
Rest (Avg Baseline)	24	1.03±0.307	1.16±0.388	0.01*
Max Cont (Avg Work)	24	41.91±25.03	47.78±33.12	0.20
Speech (Avg Work)	24	9.25±5.83	9.39±7.87	0.04*
Pain threshold	24	2.071±0.709	1.956±0.710	0.184

Avg: Average; N: Total number of participants; SD: Standard deviation; p:

* <0.05. T0: This measurement was taken in the morning before using any protective mask during the day; unmasked. T1: This measurement was taken in the evening with a mask on, when after at least five hours of mask use; with mask.

## DISCUSSION

The findings of this study indicate that prolonged use of protective masks may result in elevated stress levels, restricted mouth movements during speech, headaches, a sensation of ear pressure, and jaw joint tension. Additionally, the study concluded that prolonged mask use was associated with an increase in masseter muscle activity at rest and when speaking. Numerous clinical studies and reviews have investigated the potential issues associated with mask usage. The reported problems include increased headache, stress, oral issues, dizziness, and fatigue.^14^

A recent study found an association between wearing protective face masks for more than four hours and experiencing symptoms, such as headaches and discomfort in the ear region.^15^ The results of the study indicate that prolonged mask use, exceeding five hours, can lead to elevated stress levels, restricted mouth movements, and the onset of symptoms such as headache, ear discomfort, and jaw tension. These findings were consistent with those of previous studies.

As outlined in the literature, sEMG measurements of the masseter muscle can assist in the diagnosis of TMDs.^16^ In a study by Ginszt et al., the momentary use of a surgical mask was found to have a small effect on reducing the resting potential of the masseter muscle in healthy young women.^17^ In our study, in which we evaluated masseter muscle activity using sEMG and found that it increased during rest and speech after the use of a protective mask for more than five hours. We contend that the most significant factor contributing to the discrepancy in our findings may be attributable to the duration of mask usage, and that this may also be pertinent to the utilization of different masks.

In a separate study, it was demonstrated that myofascial pain is associated with elevated masticatory muscle activity during rest, which corroborates our findings.^18^ It is also possible that the wearing of a protective face mask, which covers the face and masticatory muscles, particularly the masseter muscle, may result in changes in masseter muscle activity. In particular, wearing a protective mask such as the N95, which fits tightly to the lower third of the face and applies continuous pressure, may cause pain and increase masseter muscle activity during TMJ movements. Recently, some authors have suggested that muscle contraction in awake bruxism may be part of the body’s defense mechanism associated with increased anxiety and stress.^19^ Our study results revealed numerical increases in masseter muscle activity, which could be linked to reported increases in stress levels and potentially associated with bruxism. Individuals with a history of TMDs may experience a greater increase in symptoms after prolonged mask use.

The literature reports that protective masks act as acoustic filters and can therefore, result in increased vocal effort required for speaking loudly.^20^ Moreover, wearing a mask could hinder the release of air during speech, causing individuals to exert more effort. The majority of participants in our study reported exerting more effort to speak loudly while wearing a mask, perhaps because they felt that their voices might not have been heard. Furthermore, the results of our study indicated that following the daily use of protective masks for an average of five hours, masseter muscle activity increased during the reading of a standard text containing speech sounds. These results are consistent with those of previous studies. Further research is required to investigate the long-term effects of protective facemasks on speech aerodynamics and potential development of voice pathology.

### Limitations:

First, the brief follow-up period and exclusive use of a single type of mask restrict the generalizability of the findings, thereby underscoring the need for future research on the long-term effects of use of different types of masks. Furthermore, the homogeneity of the sample, which consisted solely of healthy young women, limits the generalizability of the findings. Future studies should include a sample covering a wider age range, including male and female individuals with and without TMDs.

## CONCLUSION

The lack of a study that specifically investigated the effects of protective face mask use on TMJ and masseter muscle activity with sEMG in the literature makes this study a pioneering contribution to the field. This study makes a unique contribution to the existing literature by investigating the effects of using N95 masks (for at least five hours a day) on the TMJ. Furthermore, the relationship between mask use and the occurrence of physical symptoms was observed as an increase in masseter muscle activity during both rest and speech. From a clinical perspective, the results of this study offer valuable insights into whether long-term mask use may contribute to the onset of TMJ complaints or worsening of existing symptoms. We believe that these comprehensive findings serve as valuable reference for future research and clinical applications.

### Authors Contribution:

**SÖG, YY, PK, GE and EU** conceived, designed and did statistical analysis, editing of manuscript, data collection.

**SÖG and YY** participated in doing literature search, critical review, and preparing the manuscript.

SÖG did review and final approval of manuscript

All authors have approved the final version of the manuscript and are accountable for the integrity of the study.
